# Importance of a Rapid and Accurate Diagnosis in *Strongyloides Stercoralis* and Human T-Lymphotropic Virus 1 Co-infection: A Case Report and Review of the Literature

**DOI:** 10.3389/fmicb.2017.02346

**Published:** 2017-12-06

**Authors:** Olga Quintero, Carolina A. Berini, Carlos Waldbaum, Alejandra Avagnina, María Juarez, Silvia Repetto, Juan Sorda, Mirna Biglione

**Affiliations:** ^1^Servicio de Gastroenterología del Hospital de Clínicas “José de San Martín”, Buenos Aires, Argentina; ^2^Instituto de Investigaciones Biomédicas en Retrovirus y SIDA, CONICET—Universidad de Buenos Aires, Buenos Aires, Argentina; ^3^División Anatomía Patológica Hospital de Clínicas “José de San Martín”, Buenos Aires, Argentina; ^4^Instituto de Investigaciones en Microbiología y Parasitología Médica, CONICET—Universidad de Buenos Aires, Buenos Aires, Argentina

**Keywords:** strongyloidiasis, HTLV-1, Th1, corticosteroids, PCR

## Abstract

*Strongyloides (S.) stercoralis* and Human T-Lymphotropic Virus 1 (HTLV-1) share some endemic regions such as Japan, Jamaica, and South America and are mostly diagnosed elsewhere in immigrants from endemic areas. This co-infection has not been documented in Argentina although both pathogens are endemic in the Northwest. We present a case of *S. stercoralis* and HTLV-1 co-infection with an initial presentation due to gastrointestinal symptoms which presented neither eosinophilia nor the presence of larvae in stool samples in a non-endemic area for these infections. A young Peruvian woman living in Buenos Aires attended several emergency rooms and finally ended up admitted in a gastroenterology ward due to incoercible vomiting, diarrhea, abdominal pain, fever, and weight loss. Gastrointestinal symptoms started 3 months before she returned to Argentina from a trip to Peru. She presented malnutrition and abdominal distension parameters. HIV-1 and other immunodeficiencies were discarded. The serial coproparasitological test was negative. Computed tomography showed diffuse thickening of duodenal and jejunal walls. At the beginning, vasculitis was suspected and corticosteroid therapy was initiated. The patient worsened rapidly. Skin, new enteral biopsies, and a new set of coproparasitological samples revealed *S. stercoralis*. Then, HTLV-1 was suspected and infection was confirmed. Ivermectin and albendazole were administrated, until the stool sample remained negative for 2 weeks. Larvae were not observed in fresh stool, Ritchie method, and agar culture 1 week post-treatment. Although she required initial support with parenteral nutrition due to oral intolerance she slowly progressed favorably. It has been highly recommended to include a rapid and sensitive PCR strategy in the algorithm to confirm *Strongyloides* infection, which has demonstrated to improve early diagnosis in patients at-risk of disseminated strongyloidiasis.

## Introduction

The soil-transmitted nematode *Strongyloides stercoralis (S.)* is estimated to infect at least 30–100 million people. The prevalence has been increasing, mainly in Southern, Eastern, and central Europe, the Caribbean, Southeast Asia, Latin America, and sub-Saharan Africa (Buonfrate et al., 2015). The filariform larvae, which inhabit the soil, usually infect humans via skin penetration. This parasite is the only helminth able to complete its life cycle inside a single host and some larvae reinfect the host through the intestinal mucosa or perianal skin, using a process called autoinfection. The majority of patients develop a chronic, asymptomatic, or mildly symptomatic infection. Auto-infection is responsible for chronic infections with eosinophilia being sometimes the only laboratory finding, while larvae excretion fluctuates at very low levels (Concha et al., 2005). However, when auto-infection occurs, the development of disseminated strongyloidiasis is possible, especially in immunocompromised hosts like patients treated with corticosteroids, cancer patients and persons infected with human T-lymphotropic virus (HTLV-1) (Ramanathan and Nutman, 2008). In these cases, hyperinfection, characterized by gastrointestinal and pulmonary hemorrhage, and secondary bacterial infections can occur (Keiser and Nutman, 2004).

HTLV-1 is an oncoretrovirus that infects ~10 million individuals with clusters of high endemicity in certain geographic areas and ethnic groups, in particular in southwestern Japan, sub-Saharan Africa, South America, the Caribbean basin and localized areas in Iran and Australo-Melanesia (Gessain and Cassar, 2012). There are three possible transmission routes for HTLV-1: sexual transmission, mother to child transmission via breast milk, and exposure to contaminated blood. This retrovirus causes an aggressive malignancy known as adult T-cell leukemia/lymphoma (ATLL) and a progressive chronic inflammatory neurological disease named HTLV-1 associated myelopathy/tropical spastic paraparesis (HAM/TSP) (Barmak et al., 2003; Proietti et al., 2005). Adult T-cell leukemia was first identified in Japan by Takatsuki and colleagues in 1977, where a high incidence in the southwest region was observed (Takatsuki et al., 1997).

*S. stercoralis* and HTLV-1 share some endemic regions such as Japan, Jamaica, and South America and are mostly diagnosed elsewhere in immigrants from endemic areas (Robinson et al., 1994; Atsushi, 1995; Gotuzzo et al., 1999; Chieffi et al., 2000).

HTLV-1 infected carriers seem to have an increased risk of acquiring strongyloidiasis indicating a possible subclinical immunodeficiency. This retrovirus also predisposes patients to recurrent and severe infection with *S. stercoralis* (Carvalho and Da Fonseca Porto, 2004). The host defense against helminths may involve both type 1 and 2 immune responses, even though IL-4, IL-13, IgE mast cells, and eosinophils play an important role in the elimination of these parasites. Although, IL-4 and IL-10 produced by Th2 cells have the ability to down-regulate Th1 cells in HTLV-1/*S. stercoralis* co-infected patients, the virus induces a Th1 profile. Hence, IFN-γ can down-regulate Th2 cells with a diminished antiparasitic response that induces an increased risk of recurrent and severe forms of strongyloidiasis with poor response to treatment (Porto et al., 2001; Carvalho and Da Fonseca Porto, 2004). Furthermore, administration of immunosuppressive or corticosteroid therapies in patients with this co-infection may trigger potentially fatal dissemination (Fardet et al., 2006). Regarding diagnosis, it has been highly recommended to include a rapid and sensitive technique in the algorithm to confirm *Strongyloides* infection after the first negative result obtained from the fresh stool sample (e.g., screening before transplantation, initiation of steroid, immunosuppression status) when this parasitosis is suspected (Dacal et al., 2017). In Argentina, a very low cost and sensible PCR has been recently designed, which has demonstrated to improve early diagnosis in patients at-risk of severe infections (Repetto et al., 2016). Administration of ivermectin for 1 or 2 days is the treatment of choice for chronic, asymptomatic infection. For hyperinfection syndrome and disseminated infections, ivermectin is the drug of choice with albendazole as a second-line therapy, until complete eradication of the parasite is achieved (Gann et al., 1994; Mejia and Nutman, 2012).

## Case report

### Ethics statement

The patient agreed and provided written informed consent for publication of this case report and any accompanying images. Due to the observational nature of this case report, no formal ethics approval was required.

**Figure 1:**
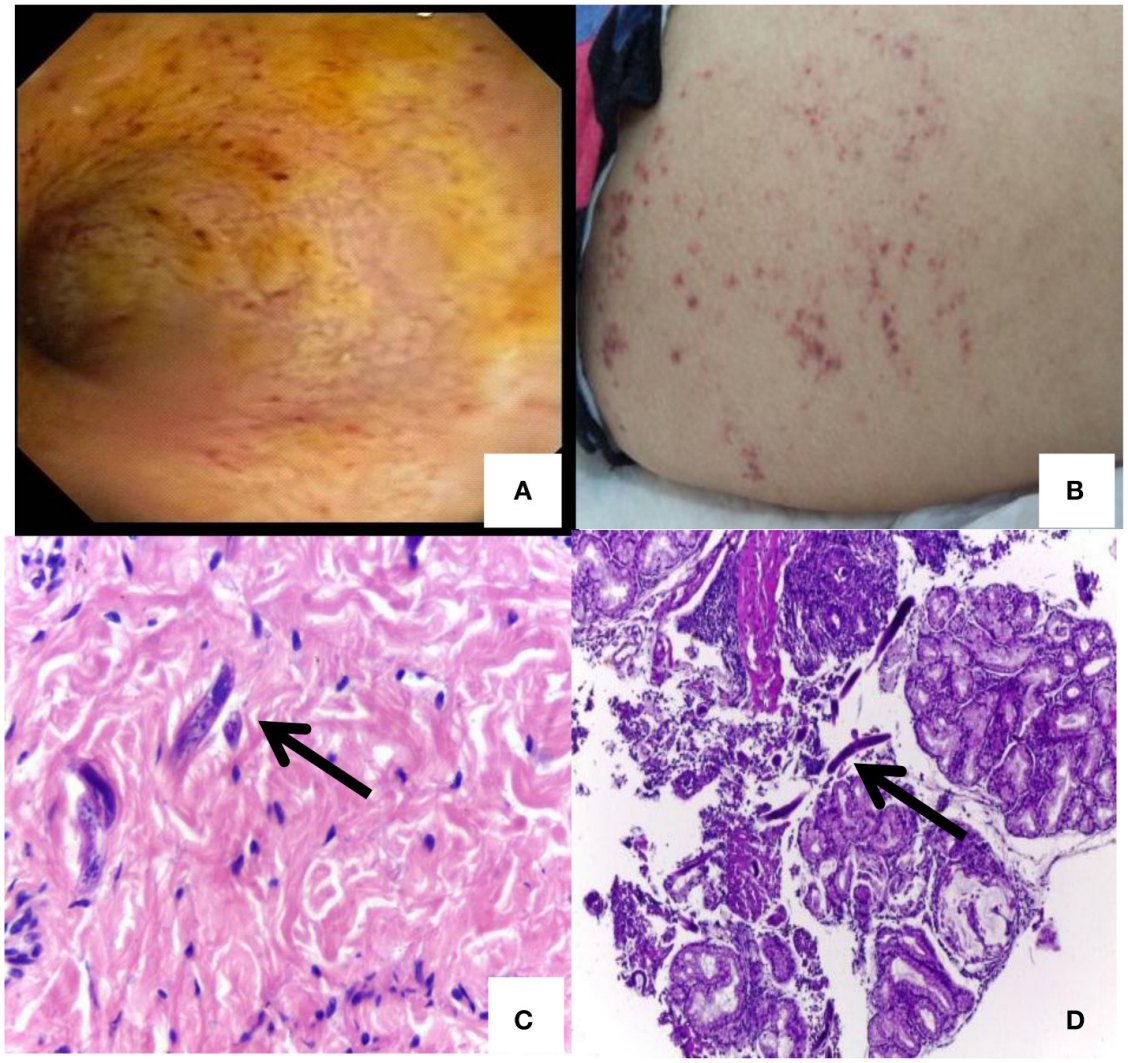
Petechial lesions and *Strongyloides stercoralis* larvae detection: **(A)** A push enteroscopy showing signs of severe duodenal and jejunal atrophy mucosa with multiple petechial lesions. **(B)** Abdominal skin rash and petechiae of disseminated *Strongyloides*. **(C)** *Strongyloides* larvae in skin. **(D)** *Strongyloides* larvae in duodenum.

A 25-year-old Peruvian woman living in Buenos Aires, Argentina attended several emergency rooms and finally ended up admitted in a gastroenterology ward of the University Clinical Hospital “José de San Martín,” in March 2016 due to incoercible vomiting, diarrhea, abdominal pain, fever, weight loss (>16 kg), and decreased visual acuity in the right eye. The patient reported that gastrointestinal symptoms had started 3 months before (December 2015) when she returned to Argentina from a trip to Peru. She presented malnutrition and abdominal distension parameters. Her admission laboratory values revealed neutrophilia (26,600/mm^3^), an increase in platelets (625,000/mm^3^), and decreased creatinine (0.44 mg/dL), sodium (111 mmol/l), potassium 5,1 mmol/l, chloride 75 mmol/l, and albumina (1.8 g/dL) values. Other values were normal and the serial coproparasitological test was negative. An inter-consultation with the ophthalmology ward was requested. HIV-1 and other immunodeficiencies were discarded. The result of a computed tomography showed diffuse thickening of duodenal and jejunal walls. A push enteroscopy and videocolonoscopy showed signs of severe duodenal and jejunal atrophy with multiple petechial lesions (Figure 1A; Video S1). Then, vasculitis was suspected and corticosteroid therapy was initiated. The patient worsened rapidly. Subsequently, urine and blood cultures detected *Escherichia coli* and *Klebsiella pneumonia*, respectively. Endophthalmitis was also diagnosed. Therefore, antibiotic treatment was indicated and slight improvement was observed. Nevertheless, the patient presented fever (38°C), abdominal pain, diarrhea stools with mucus and abdominal wall bleeding and petechiae (Figure 1B). Skin, new enteral biopsies (Figures 1C,D) and a set of coproparasitological (fresh stool and Ritchie method) samples revealed *S. stercoralis* infection. No other pathologies were found by imaging, blood, and CSF examination. Ivermectin was administrated but poor response to treatment was observed. Finally, an interdisciplinary athenaeum was organized with referents of parasitology and virology from the University of Buenos Aires.

HTLV-1 was suspected and by April 2016, anti-HTLV-1 antibodies in plasma by ELISA (HTLV I&II Ab, ULTRA version, Dia.Pro) were detected. For HTLV-1 molecular confirmation and proviral load (PVL), DNA was extracted from peripheral blood mononuclear cells (PBMCs) by column extraction (AND PuriPrep-S kit, Highway®, Inbio, Argentina) and analyzed by an “in-house” nested-polymerase chain reaction (n-PCR) and by real-time SYBR Green PCR (qPCR) respectively, as described previously (Tuke et al., 1992; Cánepa et al., 2015). HTLV-1 *pol* and *tax* regions were detected and PVL determined a total of four copies of HTLV-1/100 PBMC. After HTLV-1 detection, diagnosis was performed on her parents and they were also confirmed HTLV-1 seropositive, so mother-to-child transmission was suspected. Antiparasitic treatment, including ivermectin and albendazole were administrated, until the stool sample remained negative for 2 weeks. Larvae were not observed by fresh stool, Ritchie method, and agar culture 1 week post-treatment. She required initial support with parenteral nutrition due to oral intolerance.

## Discussion

*S. stercoralis* and HTLV-1 share some endemic regions particularly in South America and are mostly diagnosed elsewhere in immigrants from endemic areas (Carvalho and Da Fonseca Porto, 2004; Buonfrate et al., 2015). Eventhough, in Argentina, where both pathogens are endemic in the Northwest region, this co-infection has not been documented yet (Biglione et al., 2005; Repetto et al., 2016).

*S. stercoralis* patients with HTLV-1 co-infection have a modified immunological response against parasite antigens and this has clinical implications for strongyloidiasis. The high production of IFN-γ observed in co-infected patients induced by a Th1 profile results in a failure of activation of eosinophils and reduction of their numbers. This decreases the defense mechanisms against helminthes leading to an increased risk of severe forms of strongyloidiasis without eosinophilia (Porto et al., 2001) In this context, it has been hypothesized that dissemination of *Strongyloides* infection could occur due to an increase in the number of regulatory T cell (Montes et al., 2009). Otherwise, it has been observed that in this coinfection, the levels of sIL-2R are higher than those observed in patients with HTLV-1, and that anthelmintic treatment decreased them. Consequently, *S. stercoralis* infection plays a key role in the immunomodulation of HTLV-1 infection and that may be a cofactor in the development of ATLL, as reported elsewhere (Yamaguchi et al., 1987; Salles et al., 2013).

This patient had an initial presentation with gastrointestinal symptoms, normal values of eosinophils, and without detectable larvae in different stool samples. This led to symptomatic treatment without suspicion of infection in several medical wards. Moreover, and based on a first diagnosis of vasculitis when she was admitted in the gastroenterology ward, corticosteroids were administrated resulting in a severe systemic strongyloidiasis which was diagnosed by detecting larvae in different organs.

Immediately, corticosteroids were suspended and ivermectin was administrated but due to a poor response to treatment, HTLV-1 was suspected and confirmed by serological and molecular techniques. The PVL was low, similar to that of asymptomatic HTLV-1 individuals, and the patient had no symptoms associated to this retrovirus. This data indicates that low PVLs can induce a Th1 profile in asymptomatic carriers sufficiently enough to have an increased risk of developing disseminated strongyloidiasis (Porto et al., 2001). When HTLV-1 infection was confirmed, albendazole was also suggested and the patient showed a slow but favorable evolution due to the improvement in the treatment which was strictly monitored.

This case highlights the fact that all cases of parasitosis suspicion even without eosinophilia, or presence of larvae in stool samples, *S. stercoralis* should be routinely looked for by a very sensitive technique, especially in HTLV-1 individuals and patients who travel to and from endemic areas. It has been demonstrated that early and effective treatment for strongyloidiasis in HTLV-1 positive patients is important to prevent recurrent and disseminated strongyloidiasis (Salles et al., 2013). Regarding this, the use of a sensitive, economical and fast PCR technique for the confirmation of *S. stercoralis* infection, at the onset of the symptoms as proposed by Repetto et al., could have improved the early diagnosis in a patient at-risk of severe hyperinfection. This technique should also be performed for monitoring the treatment, considering it has been documented that IgG antibody levels decline within 6 months of successful treatment (Mejia and Nutman, 2012).

Another key factor to keep in mind is the epidemiological characteristic of these infections. Even though, the young woman was born and traveled to Peru, an endemic area for both pathogens, these infections were not initially suspected at different hospitals in a non-endemic region. This is frequent for HTLV-1 infection, in Argentina and other countries, where HTLV remains a “hidden” infection because this retrovirus circulates with a very low prevalence with the exception of some restricted regions and/or has not been included in a Health National Programme for epidemiological surveillance yet (Eirin et al., 2010). In this case, as her parents were from Peru and were also HTLV-1 positive, the most probable hypothesis is that she got HTLV-1 infection by vertical transmission and that she acquired *Strongyloides* in her last trip to Peru.

This case report is a useful reminder to clinicians that *S. stercoralis* as well as HTLV-1 should be considered in immigrants from endemic areas. Special attention must be given in this co-infection due to the contraindication of immunosuppressive or corticosteroid therapies and the decrease in the efficacy of anti-helmintic drugs (Satoh et al., 2002; Ramanathan and Nutman, 2008; Roxby et al., 2009). This data highlights the challenge in continuous clinical monitoring and reaching a timely and accurate diagnosis to positively impact on life expectancy.

## Author contributions

OQ, CW and JS performed the clinical diagnosis and detected *Strongiloides*’ larvae. AA and MJ provided resources, interpretation of data and performed the histopathological analysis. CB carried out HTLV-1 diagnosis. SR performed *Strongiloides*’ molecular diagnosis. All authors contributed to the interpretation of the results and provided critical feedback. MB wrote the original draft JS, CW, SR and MB realized a critical revision of the manuscript for intellectual content. CB, JS and MB reviewed and edited the manuscript.

### Conflict of interest statement

The authors declare that the research was conducted in the absence of any commercial or financial relationships that could be construed as a potential conflict of interest. The reviewer ACRV and handling Editor declared their shared affiliation.

## Supplementary material

Video S1 Push enteroscopy and videocolonoscopy of patient with Strongyloidiasis and HTLV-1 infection.
